# The Promise of Pragmatic Clinical Trials Embedded in Learning Health Systems

**DOI:** 10.5334/egems.285

**Published:** 2019-04-03

**Authors:** Leah Tuzzio, Eric B. Larson

**Affiliations:** 1 Kaiser Permanente Washington Health Research Institute, US

**Keywords:** Learning Health Systems, Pragmatic Clinical Trials

## Abstract

This commentary describes the need for a different context to clinical research that could speed the discovery and implementation of evidence-based advancements to health care delivery. Pragmatic clinical trials (PCTs) are a promising type of trial conducted within real-world health care delivery systems like organizations within the Health Care Systems Research Network, that embrace research as part of their culture of continuous learning and improvement. In these learning health systems (LHSs) clinical practice influences research and vice versa. A goal of LHSs is to operationalize evidence generated by research, particularly PCTs, into improvements that are sustained after a trial ends. PCTs that demonstrate value to health systems and foster implementation could reduce delays in translating research into practice.

## Introduction

Of the flood of research discoveries that could inform clinical practice, it has been reported that it may take up to 17 years for only 14 percent of research findings to be implemented into clinical care [1]. Production and uptake of clinical evidence is inefficient because most randomized clinical trials occur in settings that are separated from clinical care and do not always answer research questions that are relevant and meaningful to clinicians, patients, and health systems. In addition, clinicians and delivery systems are overburdened with providing routine care and addressing multiple other priorities and have little time for learning about new clinical evidence and integrating it into practice. This situation suggests that we need a different research context for generating and translating evidence into everyday practice. This commentary describes how pragmatic trials in learning health systems (LHSs) such as those in the Health Care Systems Research Network (HCSRN) provide that context.

The learning health system is a dynamic, aspirational paradigm proposed in the decades since the classic *Crossing the Quality Chasm* report [2]. The National Academy of Medicine describes LHSs as “designed to generate and apply the best evidence for the collaborative health care choices of each patient and provider; to drive the process of discovery as a natural outgrowth of patient care; and to ensure innovation, quality, safety, and value in health care” [3]. Greene et al. (2012) describe the LHS as a bidirectional relationship between health systems and researchers that aims to produce benefits by crossing the evidence-to-quality chasm, so “evidence informs practice and practice informs evidence.” Greene and coauthors describe the LHS as an ecosystem for performing effectiveness research, implementing evidence from that research, and applying the knowledge generated to improving care. To date, the impact and value of research conducted within LHSs is underreported [4,5,6,7].

### The NIH Collaboratory – Integrating pragmatic research within health systems

In 2012, the Common Fund of the National Institutes of Health (NIH) created the Health Care Systems Research Collaboratory to strengthen the national capacity to implement large-scale, cost-effective pragmatic clinical trials (PCTs) and move from explanatory trials towards more pragmatic research. Traditional, explanatory trials “are primarily designed to determine the effects of an intervention under ideal circumstance.” Whereas PCTs “are primarily designed to determine the effects of an intervention under the usual conditions in which it will be applied” [8].

The NIH Collaboratory UH3 PCTs are embedded within real-world health systems with generalizable populations, including integrated delivery systems like HealthPartners, Henry Ford Health System, and six regions within Kaiser Permanente (Georgia, Hawaii, Northern California, Colorado, Oregon, and Washington) that are members of the Health Care Systems Research Network (HCSRN) and within nursing homes, trauma centers, dialysis centers, hospitals, and community health centers (Table 1). Embedded PCTs within HCSRN and other health systems are ideal environments for PCTs because of their well-developed data infrastructure and strong collaborative links to the delivery systems. These links ensure that researchers ask questions that are relevant and important to patients, clinicians, payers and other stakeholders, and that answers to these questions are ready to be implemented by these same stakeholders. The ability to conduct PCTs depends heavily on longitudinal, electronic health record (EHR) data collected during routine care. EHR data are an efficient, rich source of information that are already generated and used in care delivery, population health management, and quality and performance analytics. Conventional wisdom, endorsed by NIH’s effort to promote PCTs, is that results of PCTs are more likely than traditional randomized controlled trials to be generalizable to everyday care settings [9].

**Table 1:** NIH Collaboratory UH3 Pragmatic Clinical Trials Embedded in Health Care Systems.

Project Title	Project Goals	Health Care Systems (HCSs)

*Strategies and Opportunities to Stop Colon Cancer in Priority Populations (STOP CRC)*	Improve the rates of colorectal cancer screening by mailing fecal immunochemical tests to patients.	26 Federally Qualified Health Centers in 3 US states (Oregon, California, and Washington)
*Pain Program for Active Coping & Training (PPACT)*	Help patients adopt self-management skills for chronic pain, limit use of opioid medications, and identify factors amenable to treatment in the primary care setting.	Primary care clinics affiliated with 3 US-based HCSs (Kaiser Permanente Georgia, Kaiser Permanente Northwest, Kaiser Permanente Hawaii)
*Time to Reduce Mortality in End-Stage Renal Disease (TiME)*	Determine whether increasing the duration of hemodialysis sessions reduces mortality and hospitalization rates for patients receiving maintenance hemodialysis care.	266 outpatient dialysis units owned by two US-based dialysis provider organizations (DaVita, Inc.; Fresenius Medical Care – North America)
*Active Bathing To Eliminate Infection (ABATE Infection) Trial*	Determine if using antiseptic bathing for hospitalized patients, plus nasal ointments for patients harboring methicillin-resistant *Staphylococcus aureus* (MRSA), reduces multidrug-resistant organisms and bloodstream infections.	53 Hospital Corporation of America hospitals in the US, including all inpatient medical, surgical, step-down, and oncology units
*Lumbar Imaging with Reporting of Epidemiology (LIRE)* *Trial*	Determine if inserting epidemiologic benchmarks into lumbar spine imaging reports reduces subsequent spine-related tests and treatments.	100 clinics in 4 US-based HCSs (Kaiser Permanente Northern California, Kaiser Permanente Washington, Mayo Clinic, Henry Ford Health System)
*Suicide Prevention Outreach Trial (SPOT)*	Compare outcomes in patients who receive care-management or online skills training for suicide prevention versus usual care.	4 US-based HCSs (Kaiser Permanente Washington, HealthPartners, Kaiser Permanente Colorado, Kaiser Permanente Northwest)
*Improving Chronic Disease Management With PIECES (ICD-Pieces)*	Improve care for patients with chronic kidney disease, diabetes, and hypertension by using a novel technology platform (PIECES) that uses the EHR to identify patients and assign practice facilitators within primary care practices or community medical homes.	4 distinct US-based HCS (Parkland Health and Hospital System, VA North Texas, Texas Health Resources and ProHealth Physicians)
*Trauma Survivors Outcomes and Support (TSOS)*	Coordinate care and improve outcomes for trauma survivors with post-traumatic stress disorder and comorbidity.	25 US-based level 1 trauma centers
*Pragmatic Trial of Video Education in Nursing Homes (PROVEN)*	Determine if showing advance care planning videos in nursing homes affects the rates of resident hospitalization.	2 US-based nursing home HCSs (Genesis, Pruitt Health) (260 nursing homes)

### How PCTs Align with Health Systems

The experience of the Collaboratory PCTs likely constitute the best-case examples to learn about the challenges of conducting research and of dissemination, implementation and sustainability of research results in real-world settings [10]. The Collaboratory PCTs start by building strong partnerships between researchers and health system stakeholders (e.g., clinicians, care team members, operational leaders, system leaders). Researchers and health system stakeholders reach an agreement to develop a research project that *aligns* with delivery system goals. Pilot testing shows if an intervention is *feasible*.

In the course of executing the trial, researchers experience the dynamic nature of modern health care. Changes in personnel, EHRs, policies and guidelines are among many issues that researchers and their delivery system partners must solve together to complete a pragmatic trial on time and on budget. Thus, establishing and nurturing trusting partnerships between researchers and health system partners is foundational. The research team from the Suicide Prevention Outreach Trial (SPOT) worked with integrated health care systems including several systems from a single corporate entity, Kaiser Permanente, but they discovered coding and EHR versions varied. They learned they couldn’t centralize the programming. Each health system needed to have a dedicated data specialist to partner with the research team to make adaptations to fit the intervention within their system. Strong partnerships require setting expectations early so research team and health system partners can work transparently and collaboratively from the beginning [11]. The Pain Program for Active Coping and Training (PPACT) research team engaged early and continuously with their delivery systems to understand how to design an intervention that would be feasible within a busy primary care setting. This partnered-research relationship was key to informing the timing of when to schedule study-related visits with patients. Another PCT, the Lumbar Image Reporting with Epidemiology trial (LIRE), benefitted from having researchers who had experience working with the health system partners and understood the EHR nuances to anticipate barriers and identify solutions that fit each health system’s context [9].

Completing a PCT requires a bidirectional flow of information and cooperative problem solving between investigator teams and clinical teams. Bidirectionality is a key feature of LHSs. This interchange involves shared knowledge of how research can relate to practice to improve health and health care. An approach to creating a collaborative learning environment is for researchers and systems partners to engage in a series of quality-improvement “plan, do, study, adjust” (PDSA) cycles in real time. This approach was used in Strategies and Opportunities to Stop Colon Cancer in Priority Populations (STOP CRC) to help refine and implement the research intervention within community health system workflows [12]. PDSA cycles are commonly used within LHS quality improvement efforts, and helpful to use in embedded research as they build skills that help clinicians and health system staff and leaders increase their capacity to adapt and sustain effective interventions.

In many PCTs, researchers and clinical system partners collaboratively build infrastructure (e.g., registries, EHR templates, operational manuals, training videos) for clinical teams to use in the trial and after the research is completed. These resources maintain engagement during the trial, and are especially helpful during long trials. The tools are referred to as “leave-behind resources,” because they are provided for operational use after study completion, to aid clinical and health system partners in maintaining the proven-effective interventions. For example, the precursor trial to the Active Bathing to Eliminate Infection (ABATE) trial developed a toolkit for the hospital intensive care unit staff to implement the intervention. The researchers found that the toolkit facilitated rapid integration of research into clinical care. Once the results of the trial were shown effective, the health system partners made the tools available across the health system. And, the research team generalized the toolkit and made it publicly available on the Agency for Healthcare Research and Quality’s web site [13]. If the ABATE Trial demonstrates positive effects and value, the health system partner plans to disseminate policy and procedures across the hospitals in their system and the researchers will publicly disseminate a generalizable toolkit.

Some PCTs find that the expected value of an intervention is not fully realized. This finding may reflect that the work is not feasible in the real world of clinical care delivery. Alternatively, the intervention may simply not be effective, suggesting that de-implementation is more appropriate than implementation. Other PCTs may find that although an intervention has value, it is not sufficient to make the effort of changing clinical practice worthwhile—the juice is not worth the squeeze. To come to this conclusion, researchers and system stakeholders must work together throughout the PCT to continuously evaluate the process of the research intervention within the health system to learn what does and does not work. These lessons can also be used to make iterative improvements to the process during the trial.

Evaluation of the process of conducting a PCT within a health care system is a key distinction of PCTs. In traditional efficacy and explanatory clinical trials there is close monitoring of fidelity to the trial protocol [8]. In PCTs, iterative, continuous evaluation and adaptation are valued. These adaptations inform how the interventions will be used in practice. Identifying facilitators and barriers and evaluating the feasibility and acceptability of aspects of the trial informs analyses about the likelihood of integrating and sustaining an intervention in the clinical system after the study ends. If an intervention is adopted, knowledge from these analyses inform how it is implemented. Thus, the experience and knowledge gained from PCTs gives decision makers evidence for deciding about programs or clinical changes to implement once the research is complete.

## Conclusion

The NIH Collaboratory PCTs provide a collaborative mechanism through which funders, researchers, and health system stakeholders can efficiently generate knowledge and implement effective solutions. These PCTs were conducted in real-world LHSs that have large and diverse populations such as those in the HCSRN where generating and adapting to new evidence of effective care is the norm. Although health care systems can and do change their improvement priorities, we believe that embedded PCTs expedite translating results into practice by first determining if a trial is feasible and if so, how its results can be put into evidence-based clinical practice. PCTs that demonstrate value to health systems and foster implementation could reduce delays in translating research into practice. We must continue this promising journey of implementing and evaluating embedded pragmatic research because using knowledge to improve care and health outcomes shouldn’t take 17 years. The major lessons from the PCTs embedded in LHSs are to use robust, longitudinal EHR data collected during routine care, develop bi-directional relationships between researchers and health system partners, and allow for flexibility and adaptability. If we can do these three things, 86 percent of our research that could benefit patients does not go to waste.

## References

[1] Balas, EA and Boren, SA. Managing clinical knowledge for health care improvement. Yearb Med Inform. 2000; 9(1): 65–70. DOI: 10.1055/s-0038-1637943 27699347

[2] Institute of Medicine (US) Committee on Quality of Health Care in America. Crossing the quality chasm: A new health system for the 21st century. Washington (DC): National Academies Press (US); 2001. 25057539

[3] Institute of Medicine (US) Roundtable on Evidence-Based Medicine. The Learning Healthcare System: Workshop summary. In: Olsen, LA, Aisner, D and McGinnis, JM (Eds.). Washington (DC): National Academies Press (US); 2007. 21452449

[4] Flum, DR, Alfonso-Cristancho, R, Devine, EB, Devlin, A, Farrokhi, E, Tarczy-Hornoch, P, et al. Implementation of a “real-world” learning health care system: Washington State’s Comparative Effectiveness Research Translation Network (CERTAIN). Surgery. 2014; 155(5): 860–6. DOI: 10.1016/j.surg.2014.01.004 24787113

[5] Greene, SM, Reid, RJ and Larson, EB. Implementing the Learning Health System: From concept to action. Ann Intern Med. 2012; 157(3): 207–10. DOI: 10.7326/0003-4819-157-3-201208070-00012 22868839

[6] Psek, WA, Stametz, RA, Bailey-Davis, LD, Davis, D, Darer, J, Faucett, WA, et al. Operationalizing the Learning Health Care System in an integrated delivery system. EGEMS (Wash DC). 2015; 3(1): 1122 DOI: 10.13063/2327-9214.1122 25992388 PMC4434917

[7] Budrionis, A and Bellika, JG. The Learning Healthcare System: Where are we now? A systematic review. J Biomed Inform. 2016; 64: 87–92. DOI: 10.1016/j.jbi.2016.09.018 27693565

[8] Thorpe, KE, Zwarenstein, M, Oxman, AD, Treweek, S, Furberg, CD, Altman, DG, et al. A pragmatic-explanatory continuum indicator summary (PRECIS): A tool to help trial designers. J Clin Epidemiol. 2009; 62(5): 464–75. DOI: 10.1016/j.jclinepi.2008.12.011 19348971

[9] Weinfurt, KP, Hernandez, AF, Coronado, GD, DeBar, LL, Dember, LM, Green, BB, et al. Pragmatic clinical trials embedded in healthcare systems: Generalizable lessons from the NIH Collaboratory. BMC Med Res Methodol. 2017; 17(1): 144 DOI: 10.1186/s12874-017-0420-7 28923013 PMC5604499

[10] Sherman, RE, Anderson, SA, Dal Pan, GJ, Gray, GW, Gross, T, Hunter, NL, et al. Real-world evidence – what is it and what can it tell us? N Engl J Med. 2016; 375(23): 2293–7. DOI: 10.1056/NEJMsb1609216 27959688

[11] Johnson, KE, Tachibana, C, Coronado, GD, Dember, LM, Glasgow, RE, Huang, SS, et al. A guide to research partnerships for pragmatic clinical trials. BMJ. 2014; 349: g6826 DOI: 10.1136/bmj.g6826 25446054 PMC4707716

[12] Coury, J, Schneider, JL, Rivelli, JS, Seibel, E, D’Agostini, B, Taplin, SH, et al. Applying the Plan-Do-Study-Act (PDSA) approach to a large pragmatic study involving safety net clinics. BMC Health Serv Res. 2017 6 19; 17(1): 411 DOI: 10.1186/s12913-017-2364-3 28629348 PMC5477281

[13] Agency for Healthcare Research and Quality. Universal ICU decolonization: And enhanced protocol. Available at: http://www.ahrq.gov/professionals/systems/hospital/universal_icu_decolonization. Accessed 26 November 2018.

